# Comparison of early-stage changes of osteoarthritis in cartilage and subchondral bone between two different rat models

**DOI:** 10.7717/peerj.8934

**Published:** 2020-04-20

**Authors:** Yutao Yang, Peiran Li, Songsong Zhu, Ruiye Bi

**Affiliations:** State Key Laboratory of Oral Diseases, National Clinical Research Center for Oral Diseases, Department of Oral & Maxillofacial Surgery, West China Hospital of Stomatology, Sichuan University, Chengdu, Sichuan, China

**Keywords:** Osteoarthritis (OA), Naturally-occurring OA, Secondary OA, ACLT, Collagenase, Subchondral bone, Cartilage

## Abstract

Osteoarthritis (OA) is a chronic degenerative joint disease and the major cause of joint pain and disability in the elderly. It is mainly characterized by articular cartilage degradation and subchondral bone remodeling. There are two main types of OA: natural occurring OA and secondary OA, mainly associated with aging and trauma, respectively. In this study, we established two OA models in rat knee joints to simulate the two types of OA, using the type II collagenase injection (CI) and anterior cruciate ligament transection (ACLT), respectively. After intervention for 2–6 weeks, cartilage and subchondral bone changes were detected in histological staining, immunochemistry, and micro-CT. Results showed that both models with typical pathology changes of OA were successfully induced, while the development and severity of OA process in the models were different. In ACLT rats, the cartilage damage was milder, lasted for a shorter time, and subchondral bone reconstruction occurred earlier, compared with the changes in CI rats. The cartilage damage was secondary to subchondral bone change in ACLT rats, while subchondral bone change was secondary to cartilage degeneration in CI rats. In conclusion, the interaction between cartilage and subchondral bone is different between the natural-occurring and secondary OA models. These two models not only suggest potential different mechanisms of the two types of OA, but also provide new directions for OA treatment and prevention.

## Introduction

Osteoarthritis (OA), as a degenerative joint disease, is one of the leading causes of disability in the elderly (Glyn-Jones et al., 2015). It is characterized by cartilage degradation, subchondral bone remodeling, osteophyte formation, and dysfunction of synovial membrane and surrounding structures (Lohmander, 2000). OA can be classified into two main types based on etiology: the natural occurring OA and the secondary OA. The natural occurring OA, or primary OA, is diagnosed in the absence of any predisposing event, but is associated with risk factors, especially age (Varela-Eirin et al., 2018). The secondary OA is associated with certain inducing factors, especially trauma (Kuyinu et al., 2016). Many details of OA pathogenesis of human beings still remain unknown in clinical practice. Few studies have focused on different mechanisms between the natural occurring and secondary OA.

Previous investigations have revealed a complex interaction between cartilage and subchondral bone of OA, but the triggering factor and mechanism are still debatable (Oettmeier, Abendroth & Oettmeier, 1989). Some studies suggested that the degradation of cartilage was secondary to sclerosis of subchondral bone (Sanchez et al., 2012), and that delivery of drug to prevent the sclerosis of subchondral bone can relieve the destruction of the cartilage (Bendele, 2001). However, some studies held opposite views, suggesting that the cartilage may have an effect on the subchondral bone (Li et al., 2019). In fact, we consider the contradiction is probably due to the difference in different types of OA models, and the interactions between the cartilage and bone probably differ in different models. Therefore, we made this study to deeply explore the mechanisms of OA.

The induction of OA models mainly includes surgical and drug/chemical methods. And the knee is the best described and the most frequently used joint (Kuyinu et al., 2016). The surgical procedures mainly include medial meniscal tear, partial meniscectomy, total meniscectomy, anterior or posterior cruciate ligament transection (ACLT or PCLT), and ovariectomy, etc. The chemical models, carried by intra-articular injection of chemical agents, mainly include monosodium iodoacetate, collagenase, and papain, etc. (Lampropoulou-Adamidou et al., 2014; Gregory et al., 2012). First, the type II collagen is the main component of cartilage matrix of knee joints, and naturally-occurring OA patients show chondrocyte apoptosis and degeneration of type II collagen. Previously, a few studies have revealed the similarity of articular cartilage lesion between naturally-occurring OA and the intra-articular injection of type II collagenase (Hong et al., 2014; Park et al., 2018; Yeh et al., 2008). Therefore, we conducted the collagenase injection to simulate the naturally-occurring OA. Second, injury to the anterior cruciate ligament is a risk factor for knee OA. The anterior cruciate ligament transection (ACLT) leads to imbalance of stress system, and induces joint changes analogous to those observed in post-traumatic human OA (Ferrándiz et al., 2014). So we conducted ACLT to simulate the secondary type of OA. Therefore, we used CI and ACLT as the representative models to explore the different pathogenesis between the two main types of OA.

The early-phase pathological lesions mainly include cartilage degradation and subchondral bone remodeling. Previous studies indicated that the incidence of osteophyte in rats take about 10 weeks after surgery; at that time, OA has progressed to the advanced phase and all the models would show severe lesion (Hayami et al., 2004). Because our study focused on the difference of pathogenesis between the two representative models, we only observed early-phase changes and set the period as 2–6 weeks.

The objectives of this study are: (1) to observe the early-stage changes (2–6 weeks) in articular cartilage and subchondral bone in the two models; (2) to explore the mechanisms of aging-related primary OA and traumatic-related secondary OA; (3) to provide more information for potential treatment and future research on OA.

## Materials & Methods

### Animals

Seventy-two adult male Sprague-Dawley rats (10 weeks old, 260–280 g) were provided by the Laboratory Animal Center of Sichuan University. Animals were randomly allocated into four groups (*n* = 18 per group): anterior cruciate ligament transection group (ACLT), collagenase-injection group (CI), sham-operated (Sham) group, and saline group (placebo group). The animals were housed under a 12-hour our light-dark cycle with free access to food and fresh water at room temperature. The protocol was approved by the Ethics Committee of West China Hospital of Stomatology, Sichuan University (Institutional Review Board approval number: WCHSIRB-D-2017-079).

### Induction of OA

The ACLT model was induced as previously described (Stoop et al., 2000). Briefly, rats were generally anesthetized with intraperitoneal injection of sodium pentobarbital. After disinfection, the knee joint was exposed through a medial parapatellar approach. The patella was dislocated laterally, and the knee joint was fully flexed. After that, the ACL was exposed and completely transected (Stoop et al., 2001). After being washed with sterile saline, the joint capsule and subcutaneous tissue were closed using 4-0 absorbable suture. Skin was closed with 3-0 Nylon threads. The rats in the sham-operated group were dealt with the same procedures except cutting the ACL.

The rats in the CI group were anesthetized in the same way as in the ACLT group. The type II collagenase (Sigma, USA) solution was dissolved in saline, and injected into the joint capsule of right knee at 0.025 ml (about 200 U) from the medial side. The rats in the Saline group were injected with equivalent volume of saline (Lee et al., 2009).

Finally, to prevent infection, all the animals were given 200,000 U benzylpenicillin potassium through intramuscular injection after surgery. 24 rats (6 rats per group) were sacrificed at week 2, 4 and 6 post-operatively. Samples of articular cartilage and subchondral bone of each knee were collected for further studies.

### Micro-CT

Specimens were investigated by a micro-CT scanner system (Scanco Medical *μ*-CT 50, Switzerland). Scans were performed using the following parameters: voltage, 55 kV; current 145 mA; integration time, 500 ms; voxel size, 18 mm. After scanning, we made both 3D reconstruction and calculation of osteogenesis property. The volume of interest (VOI) was defined as the tibia metaphysis. To distinguish the calcified tissue from the non-calcified tissue, the threshold was set as 250–700. The following parameters were calculated: the trabecular bone volume per total volume (BV/TV), mean trabecular separation (Tb.Sp), and trabecular thickness (Tb.Th) (Waarsing, Day & Weinans, 2004). BV/TV was defined as the ratio of trabecular bone volume and inner cortical bone of the total volume, and it reflected subchondral bone resorption. Tb.Sp was the mean trabecular separation, and Tb.Th was defined as the trabecular bone thickness.

### Histology

Samples were fixed in 4% paraformaldehyde for 48 h immediately after micro-CT evaluation. After dissection of muscle, the whole knee joints were decalcified in ethylene diamine tetraacetic acid (EDTA) solution at 37 °C for two weeks. Then, they were dehydrated in graded ethanol, cleared in toluene, embedded in paraffin, and cut into 4-µm serial sections. Samples were stained with hematoxylin and eosin (H&E) and toluidine blue. Histologic grading of the articular cartilage and subchondral bone were performed according to the modified mankin scoring system (Table 1) (Little et al., 1997; Wenz et al., 2000; Pritzker et al., 2006). In addition, as the tartrate-resistant acidic phosphatase (TRAP) is the biomarker of the osteoclasts, we have quantified the osteoclast number of the subchondral bone using TRAP staining. TRAP was detected using a commercial kit (Servicebio, China), following the manufacture’s instruction. All evaluations were performed in a blinded way by two independent observers.

**Table 1:** Modified mankin histological grading system of articular cartilage.

Surface	Score	Criteria
Toluidine blue staining	0	Normal
	1	Slight loss in staining (superficial zone)
	2	Moderate decrease in staining (mid zone)
	3	Large decrease in staining (deep zone)
	4	No staining
Cartilage integrity	0	Normal
	1	Discontinuity at cartilage surface
	2	Cartilage surface damaging and vessel erosion
	3	Clefts at superficial zone
	4	Clefts extending to mid cartilage
	5	Clefts extending to deep zone
	6	Full-thickness cartilage defect
Tidemark	0	Continuous
	1	Discontinuity, crossed with blood vessels
Arrangement of chondrocytes	0	Normal
	1	Hypercellularity
	2	Cloning
	3	Hypocellularity
Total	0–14	

### Immunohistochemistry

The expression of markers related to inflammation, cartilage matrix degradation and bone mineralization was chosen for immunohistochemistry staining. Briefly, primary antibodies to MMP13, Col II and Rux2 were purchased from Abcam (UK), and the secondary antibody immunochemistry kit was bought from Zsbio (China). After deparaffinization and hydration with toluene and gradual ethanol, tissue sections were immersed in buffer solution and heated in microwave oven for 5 min to repair antigen. The sections were incubated with 3% H_2_O_2_ to inhibit endogenous peroxidase, and then blocked with goat serum solution. After that, the sections were incubated with rabbit anti-rat primary antibodies at proper dilution at 4 °C overnight. After being washed with PBS for 3 times, the tissue sections were incubated with biotinylated goat anti-rabbit secondary antibody at room temperature for 30 min, and then with horseradish peroxidase streptavidin-albumen (S-A/HRP) for 30 min. Proteins were visualized by DAB solution, and nucleus was stained with hematoxylin. The positive cells were calculated using Image plus (USA).

### Statistical analysis

Data are presented as mean ± SD. Statistical analysis was performed by mixed-design analysis of variance (AVOVA) using SPSS 20.0, in analysis of micro-CT, TRAP staining and immunohistochemistry. Through this method we could analyze not only the comparison between the four groups, but also the time-dependent changes in each group. Rank sum test (Kruskal-Wallis test) was used in analysis of modified mankin scores as they were non-continuous variables. *P* < 0.05 was considered significant.

## Results

### Micro-CT evaluation of early subchondral bone changes during OA progression

**Figure 1:**
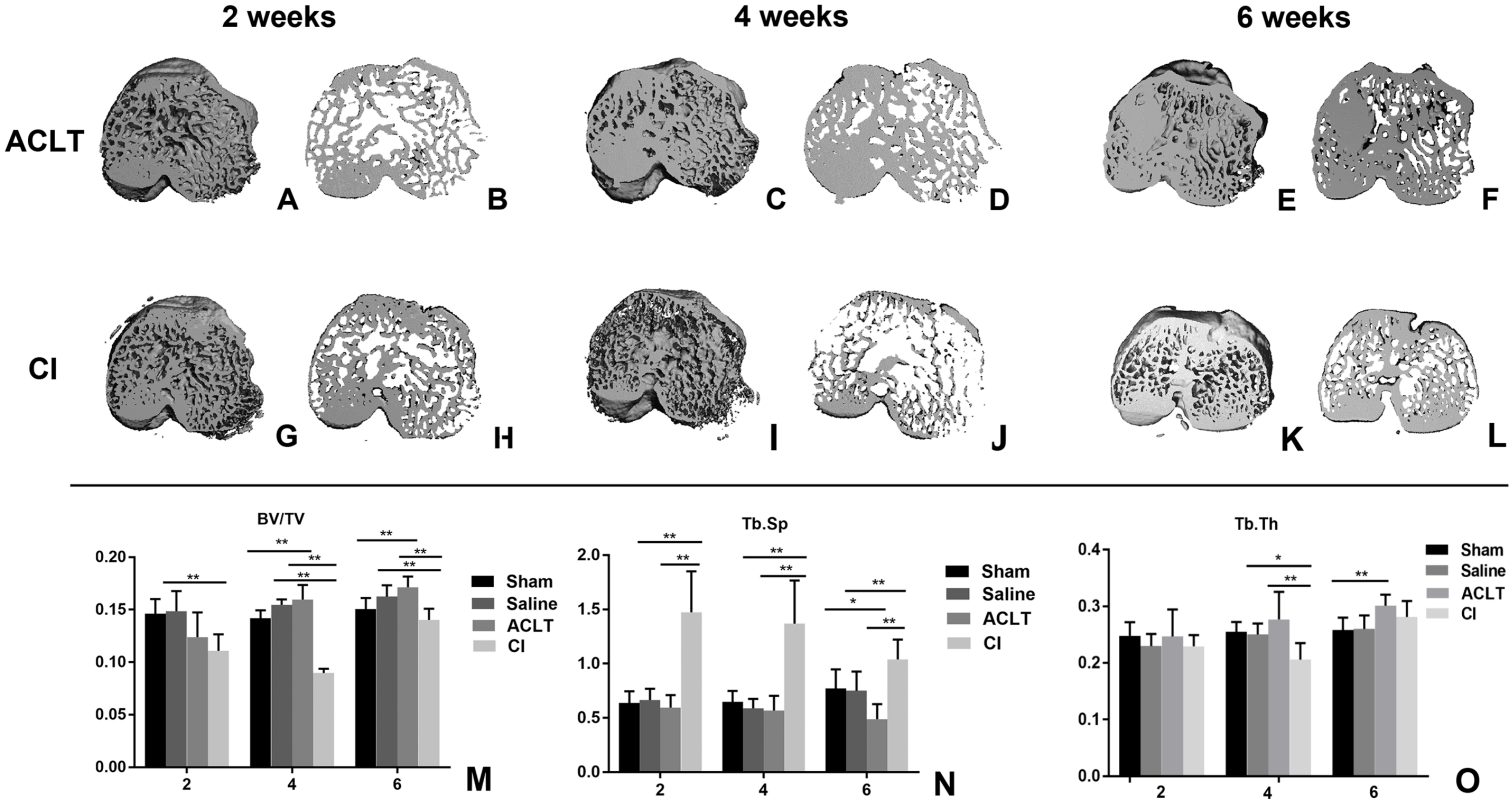
Micro-CT reconstruction and quantitative analysis of the subchondral bone of ACLT andCI models. (A–F) Micro-CT reconstruction of the subchondral bone of ACLT rats after 2, 4 and 6 weeks; (G–L) micro-CT reconstruction of the subchondral bone of CI rats after 2, 4 and 6 weeks; (M–O) micro-CT quantitative analysis of the subchondral bone after 2, 4 and 6 weeks (mean ± SD). (M) BV/TV, the subchondral trabecular bone volume per total volume. (N) Tb. Sp, mean trabecular separation. (O) Tb. Th, trabecular thickness. *Statistically significant, *P* < 0.05. **Statistically significant, *P* < 0.01.

As shown in Fig. 1, bone volume in the ACLT group decreased at the first 2 weeks and gradually increased at the 4th and 6th week (2w vs. 4w, *P* < 0.001; 2w vs. 6w, *P* < 0.001; 4w vs. 6w, *P* < 0.001), whereas bone volume in the Sham group did not show significant change in the whole period (2w vs. 6w, *P* > 0.05). For the comparison between ACLT and Sham group, it was significantly different at the 4th and 6th week (*P* = 0.002, 0.002). These results were similar in the CI and Saline group. For the comparison between the ACLT and CI group, we found that the decrease of bone volume in CI model was more severe and lasted for a longer period (4 weeks) than that in ACLT model (2 weeks). Significant difference was found between ACLT and CI groups 4 or 6 weeks after surgery (*P* < 0.001), but not significant between them after 2 weeks (*P* > 0.05). The tendency of Tb.Th and Tb.Sp was almost in consistence with that of BV/TV. With regard to Tb.Th, the mean for CI group is 0.230, 0.206 and 0.282, and for ACLT group is 0.247, 0.277 and 0.301 after 2, 4 and 6 weeks respectively. Both ACLT and CI group showed significant increase during the whole process (2w vs. 6w, *P* < 0.001), while that in Sham group did not show significant change (*P* > 0.05). Significant difference was found between ACLT and CI groups after 4 weeks (*P* = 0.001), while no significant difference after 2 or 6 weeks (*P* > 0.05). With regard to Tb. Sp, the mean for CI group is 1.473, 1.370 and 1.040, and for ACLT group is 0.595, 0.569 and 0.489 after 2, 4 and 6 weeks respectively. Significant difference was found between ACLT and CI groups in the whole period (*P* < 0.001).

### Histological analysis

**Figure 2:**
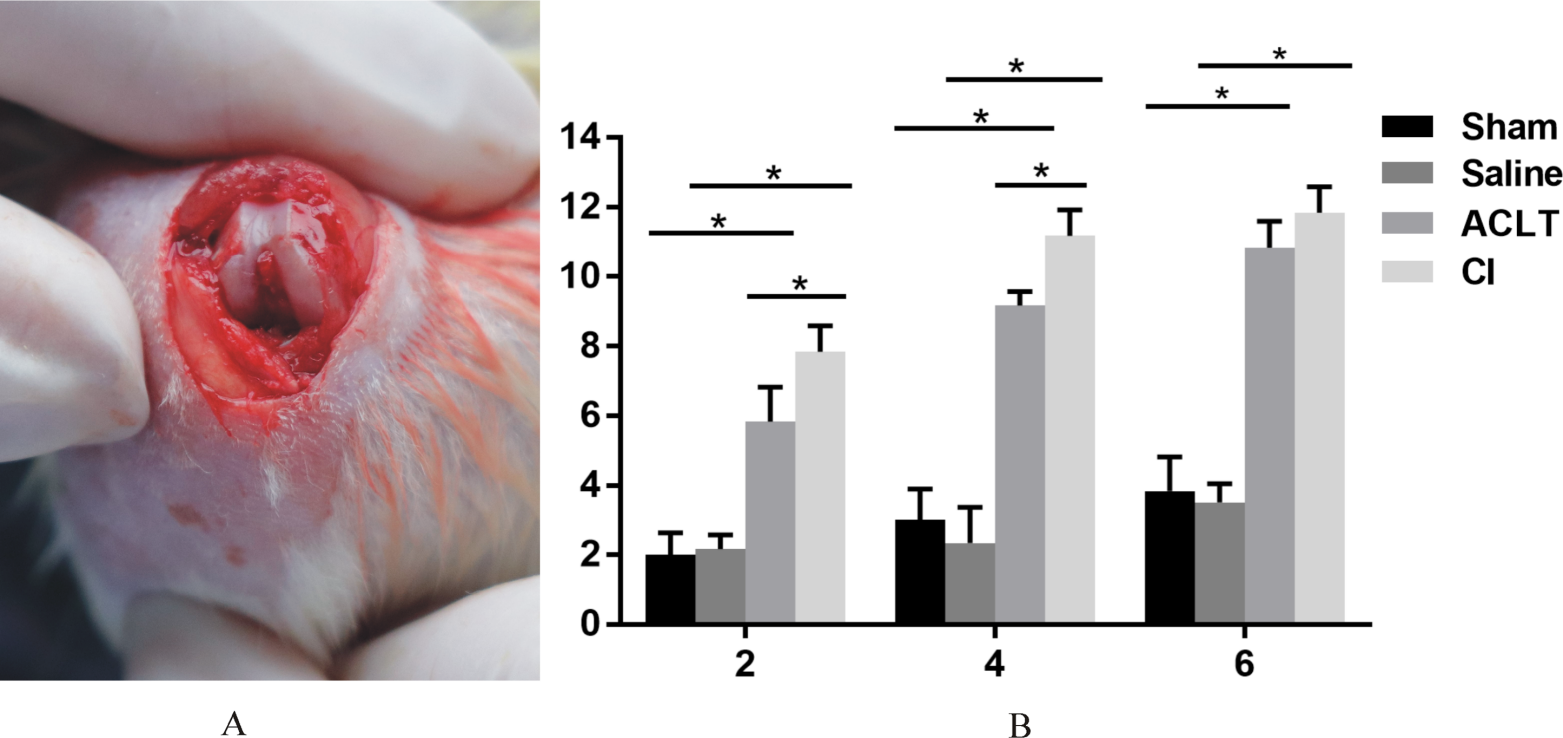
The surgical procedure and modified mankin scores. (A) The induction of OA by ACLT surgery. (B) Modified mankin scores of articular cartilage damage in four groups after 2, 4 and 6 weeks (mean ± SD). *Statistically significant, *P* < 0.05.

**Figure 3:**
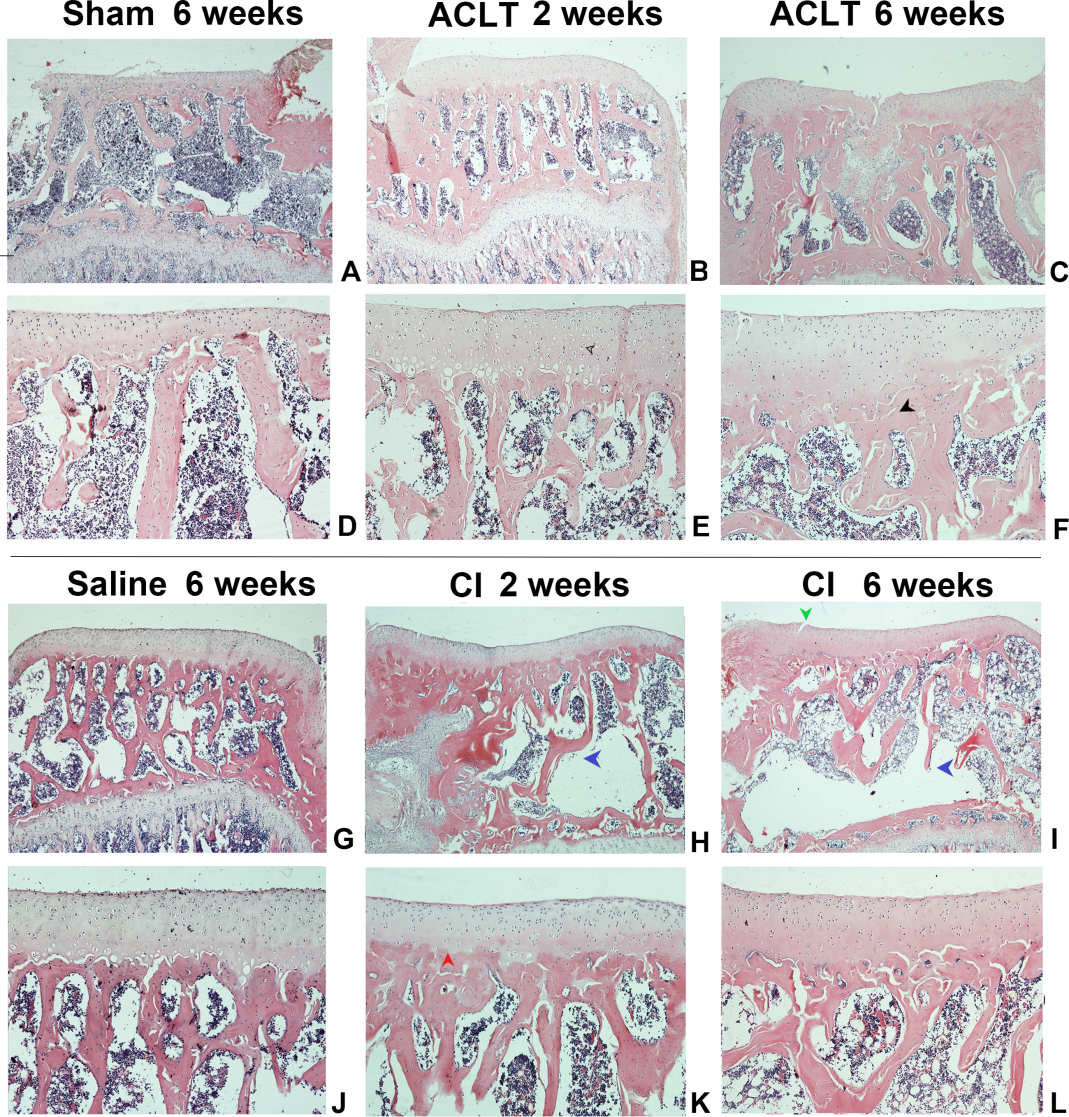
The histological assessment (HE staining) of the ACLT and CI group. Matrix fissure and irregular chondrocyte arrangement were observed as early as 2 weeks in CI group after injection (H, I), while that occurred until 6 weeks after ACLT surgery (F). The bone resorption was more severe and lasted for a longer time in CI rats (H, I, K, L), while that in ACLT rats was milder, last for a shorter time, and subsequently reconstructed at an early time (B, C, E, F). For the Sham or Saline groups, the articular cartilage surface was smooth and chondrocytes were regularly arranged at the whole period (A, D, G, J). Red arrow, tidemark; green arrow, cleft; black arrow, subchondral bone sclerosis; blue arrow, subchondral bone resorption; hollow arrow, cartilage and chondrocytes.

**Figure 4:**
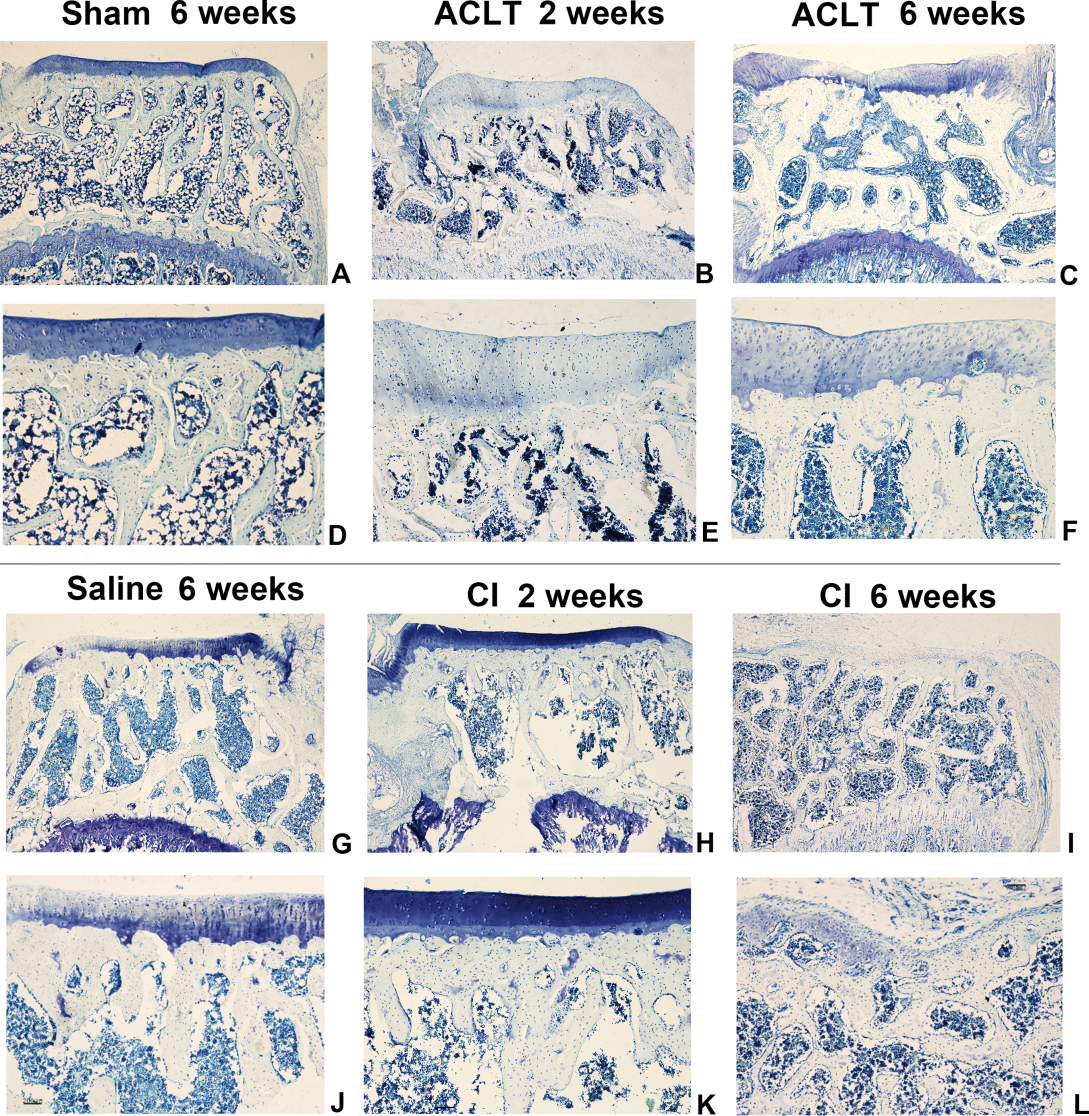
The histological assessment (toluidine blue) of the ACLT and CI group. Articular cartilage displayed a more severe matrix loss in CI rats at the 6th week than that in ACLT rats (I, L). Matrix fissure of cartilage was observed as early as 2 weeks in CI group after injection (H, K). For the Sham or Saline groups, the toluidine blue staining was almost homogeneous at the whole period (A, D, G, J).

We found a significant cartilage erosion and subchondral bone remodeling in both ACLT surgery group and CI group, while the sham operation group and saline injection group showed normal morphology of cartilage, chondrocytes and bone at any time after intervention. We also found that the orders of the lesions in cartilage and subchondral bone were different in the ACLT and CI models. The modified mankin score was shown in Fig. 2, and the histological staining was shown in Figs. 3 and 4. For CI group, matrix cleft could be observed as early as 2 weeks after intervention. Cartilage defect was more severe in CI rats at the 6th week than that in ACLT rats. For ACLT group, however, matrix cleft and irregular arrangement of chondrocytes could not be observed until the 6th week. Subchondral bone resorption lasted for a shorter time and the reconstruction occurred earlier in ACLT rats than that in CI rats.

The results of modified mankin scores also revealed the differences between the ACLT and CI groups. The score of both treated groups started to be significantly higher compared with that of Saline or Sham models 2 weeks after surgery, and lasted for the whole observing period (*P* < 0.05). And the score was also significantly higher in the CI model than that in the ACLT model after 2 and 4 weeks (*P* < 0.05). After 6 weeks, both ACLT and CI rats manifested severe cartilage damage and high mankin scores, while no difference was found between them (*P* > 0.05) (Fig. 2).

**Figure 5:**
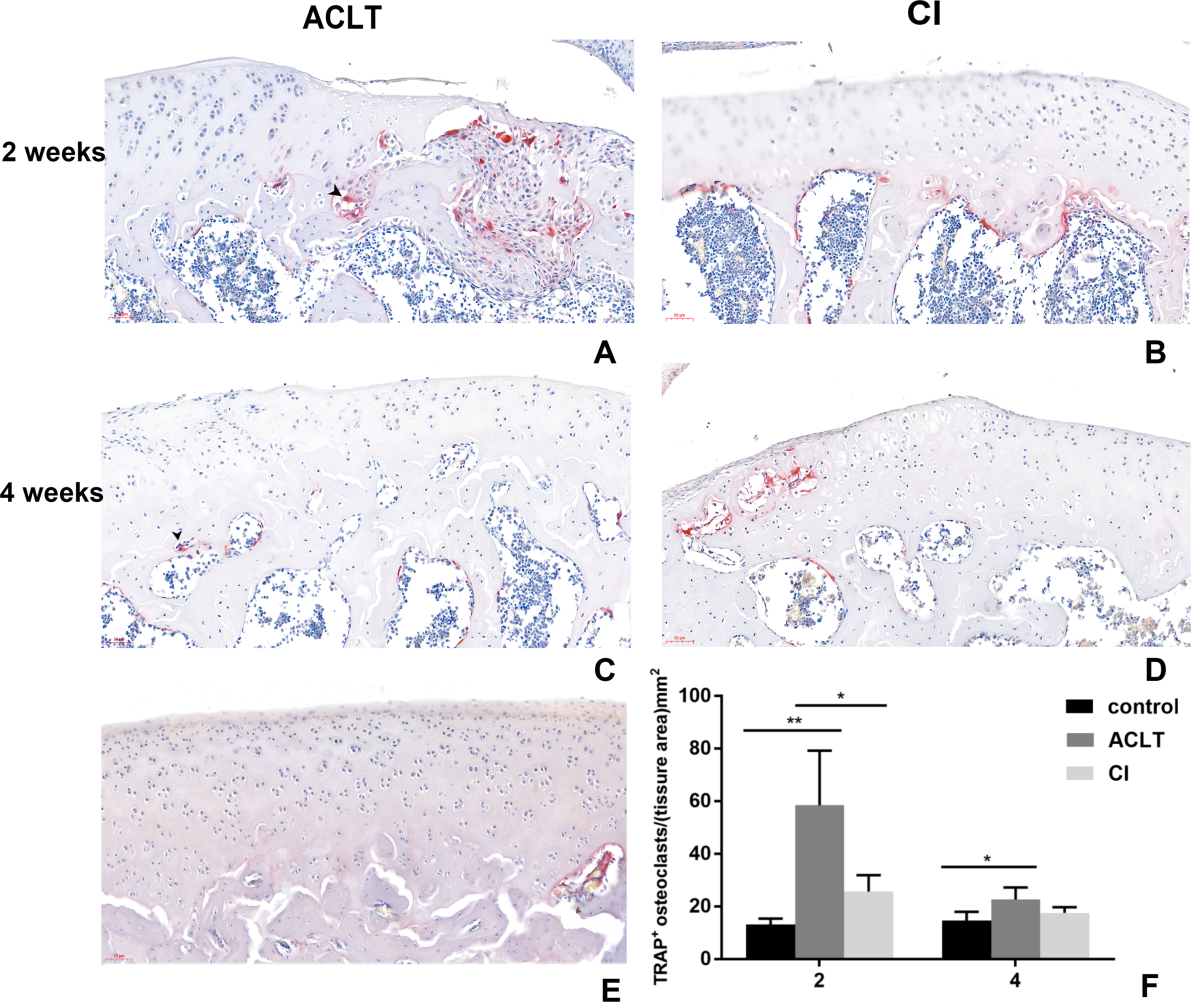
TRAP staining in ACLT and CI rats at the 2nd and 4th week. Osteoclasts were stained red, as the black arrow showed (A, C). Osteoclast activity in the subchondral bone in both two models increased at 2 weeks following treatment, but then reduced after 4 weeks (compare between A and C, B and D; and F). Significant difference was found between ACLT and CI after 2 weeks (F), which indicated that initial bone resorption may started earlier in ACLT rats. TRAP positive osteoclasts were rare in control group (E). *Statistically significant, *P* < 0.05. **Statistically significant, *P* < 0.01.

The results of TRAP staining was shown in Fig. 5. Osteoclast activity in the subchondral bone in both groups increased at 2 weeks following treatment, compared with the control group, but then the number of positive osteoclasts in both groups reduced after 4 weeks. Significant difference was found between ACLT and CI after 2 weeks (*P* = 0.018). These results indicated that both two OA models showed significant bone absorption in the first two weeks. And we supposed that the initial bone resorption may started earlier in ACLT than in CI group.

### Immunohistochemistry

**Figure 6:**
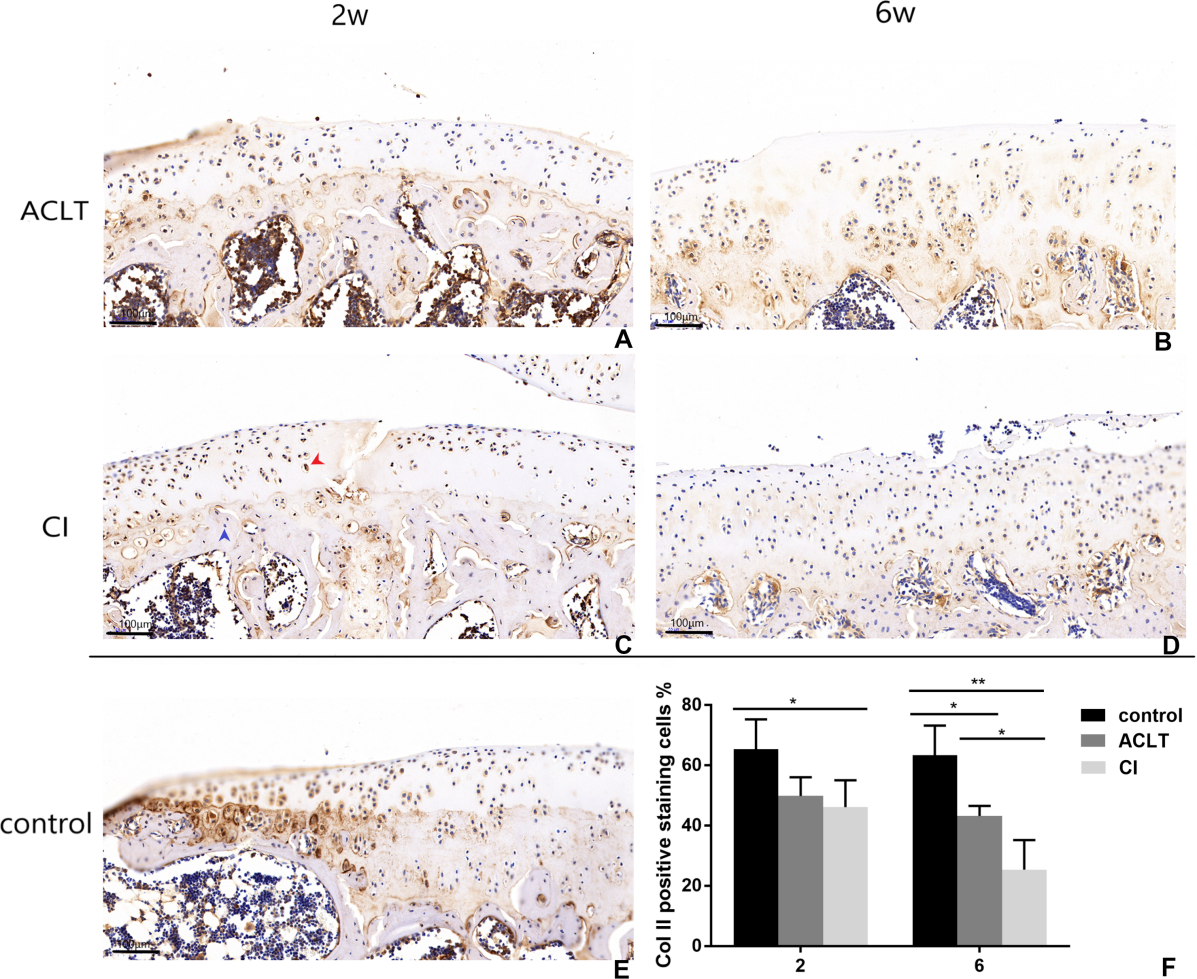
Immunohistochemical staining of Col II in ACLT and CI rats at the 2nd and 6th week. Positive cells were stained brown (as shown by the red arrow), and negative staining was marked with a blue arrow (C). Col II positive expression in articular cartilage of both groups decreased over time (compare between A and B, C and D; F), while there was more positive expression in the control group (E; F). For the comparison between ACLT and CI rats, the cartilage surface damage was more severe and the positive expression decreased to a lower level in the CI group than that in the ACLT group (compared between A and C, B and D; F). *Statistically significant, *P* < 0.05. **Statistically significant, *P* < 0.01.

**Figure 7:**
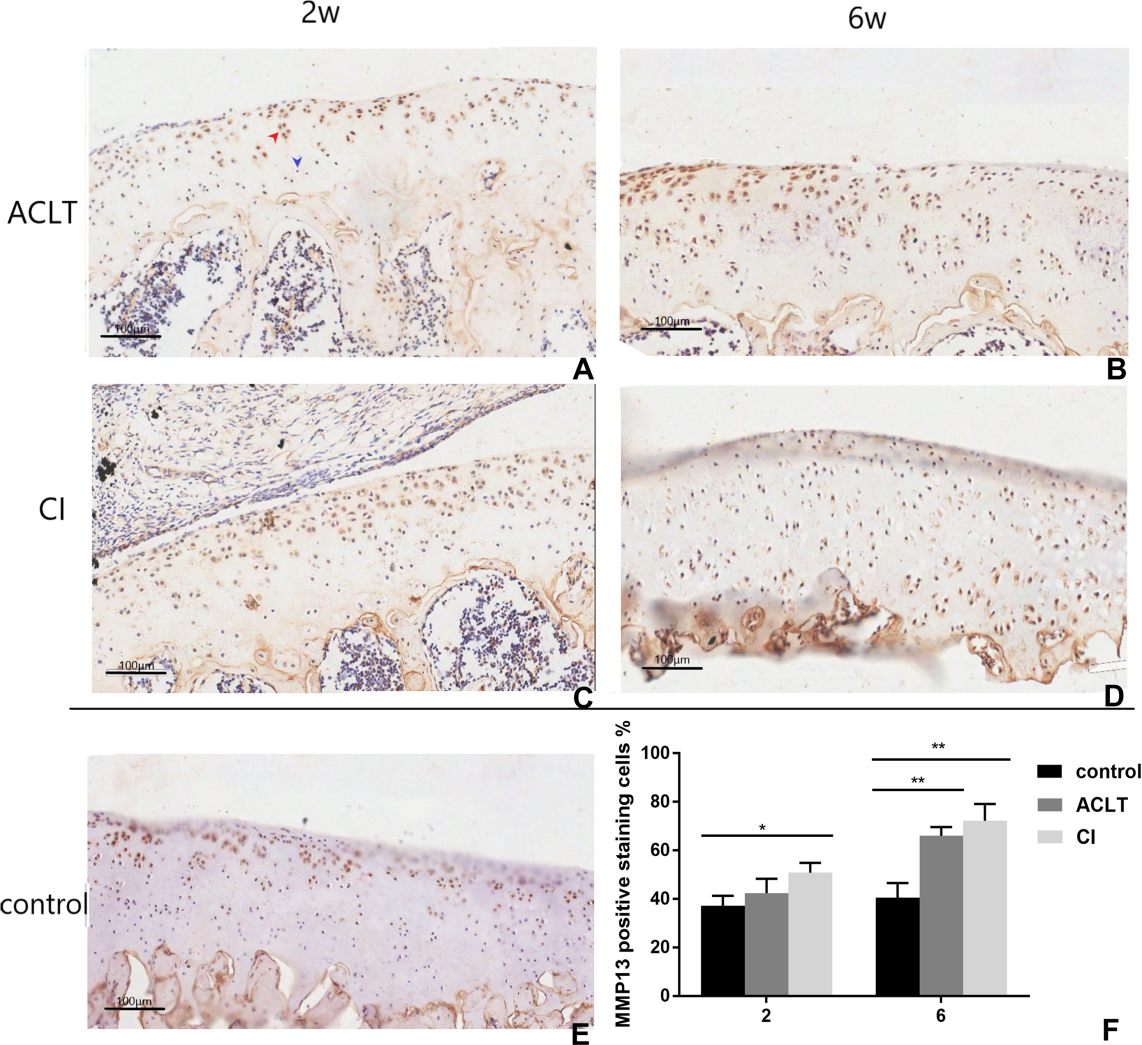
Immunohistochemical staining of MMP13 in ACLT and CI rats at the 2nd and 6th week. Positive cells were stained brown (as shown by the red arrow), and negative staining was marked with blue arrow (A). In the control group, only a few positive expression points were detected in upper region of the articular cartilage (E). For ACLT and CI rats, the positive expression significantly increased compared with the control group (B, C, D and F), and that in CI rats seemed higher than that in ACLT rats (compared between A and C, B and D). Especially, the positive expression could be detected in the full-thickness cartilage in CI rats at the 6th week (D). *Statistically significant, *P* < 0.05. ** Statistically significant, *P* < 0.01.

**Figure 8:**
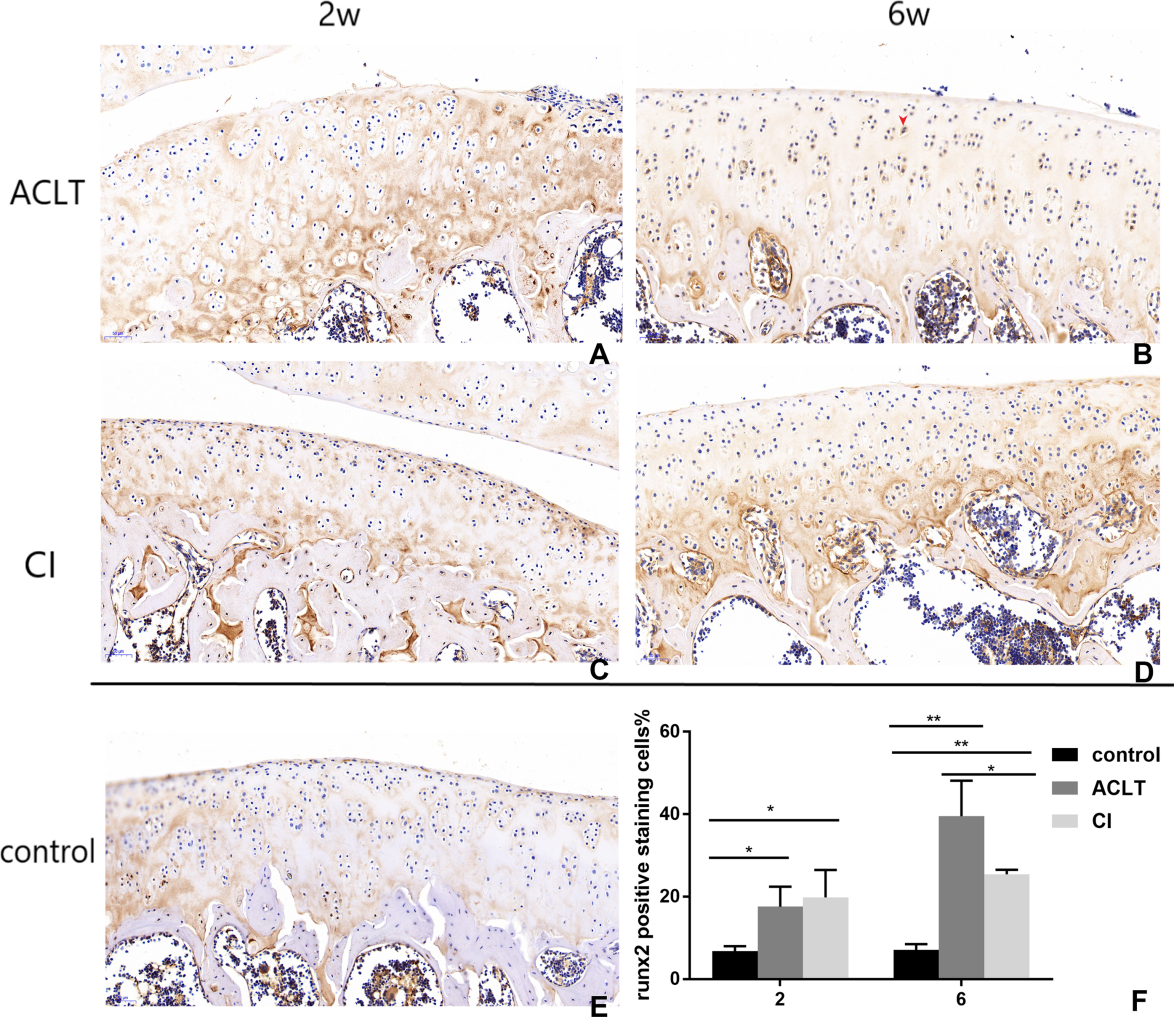
Immunohistochemical staining of Runx2 in ACLT and CI rats at the 2nd and the 6th week. Positive cells were stained brown (as shown by the red arrow) (B). They were mainly in calcified cartilage region (between the tidemark and subchondral bone). After 2 weeks, the expression of Runx2 in both ACLT and CI rats significantly increased (A, C, F), compared with the control group (E). At the 6th week, positive expression in ACLT group was significantly higher than that in CI group (B and D, F), suggesting that bone reconstruction may start earlier in ACLT rats than in CI rats. *Statistically significant, *P* < 0.05. **Statistically significant, *P* < 0.01.

Articular cartilage erosion and chondrocytes apoptosis is imbalance of synthesis and degeneration of the extracellular matrix (ECM), an event marked by the expression of proteolytic enzymes such as MMP13 and molecular of TGF- *β*/MMP 13 signaling (Wang et al., 2018). An increased expression of MMP13 could be detected in both groups from 2 to 6 weeks (*P* = 0.002, 0.004), while that change was not significant in control group (*P* > 0.05). And the positive staining of CI rats seemed to be more extensive, extending to full-thickness of cartilage after 6 weeks. Chondrocyte and cartilage matrix markers (Col II) decreased in both two groups in the whole period from 2 to 6 weeks, while in control group the change is not significant (*P* > 0.05). And cartilage damage was more severe in CI rats than that in ACLT rats after 6 weeks (*P* = 0.039). Moreover, the expression of Runx2 increased from 2–6 weeks in both treated groups (*P* < 0.001, 0.084), whereas the change is not significant in the control (*P* > 0.05), suggesting that the osteogenesis probably occurred at the early phase. The matrix mineralization region was significantly larger in ACLT rats than that in CI rats at the 6th week (*P* = 0.014), suggesting that osteogenesis in ACLT rats might start earlier (Figs. 6, 7 and 8).

## Discussion

This study aims to induce two typical OA models with different pathogenesis, to explore the histopathological differences between the two models in cartilage and subchondral bone at the early phase, and to explore more information for clinical treatment and prevention of the disease. Our study has demonstrated the histopathological difference between the two models, indicating that the pathogenic mechanisms of them were probably different.

### Pathological lesions of cartilage and bone in ACLT model and potential mechanisms

ACLT is a well-established model for OA. It simulates the whole pathological process in human joints, including cartilage degeneration, subchondral bone reconstruction, and osteophyte formation (Kuyinu et al., 2016). During the surgical procedure, the cartilage would not be disturbed. The mechanism of subchondral bone damage is associated with joint instability and overload, that is, joint stability was initially broken after transection of anterior cruciate ligament; after that, the joint activity and local stress imbalance lead to increased load on the subchondral bone. Due to the increased load, osteoclasts below the cartilage are activated, and induced subchondral bone loss (Botter et al., 2011). Actually, except for the increased number and activity of osteoclasts, our study has also demonstrated the increased level of local factors secreted by osteoblasts, such as MMP-13, which was reported to also promote the absorption of subchondral bone (Alcaraz et al., 2010; Wang et al., 2018).

Cartilage destruction in ACLT models is secondary to subchondral bone damage, we made this speculation based on our results: (1) the surgery process of ACLT would not affect articular cartilage, that is, the cartilage would not be directly damaged; (2) IHC results of collagen II showed significantly milder destruction of cartilage matrix in ACLT group than that in CI group at the 6th week, and significantly increased expression of Runx2 in ACLT group than that in CI group at the 6th week; (3) micro-CT results showed a shorter time of bone mass loss and an earlier reconstruction of subchondral bone in the ACLT model. Bone resorption lasted for 2 weeks and subchondral bone sclerosis started at the 4th week. Therefore, we supposed that bone resorption is the initiating factor for cartilage destruction in ACLT model. And the mechanisms of cartilage destruction are probably: (1) changes in density and structure of the subchondral bone induced shear stress on the cartilage above, and caused local mechanical damage on cartilage (Creaby, Wrigley & Bennell, 2007; Sanchez et al., 2012); (2) the absorbed and sclerotic subchondral bone cannot provide sufficient nutrition for the chondrocytes above, which also promote degenerative changes of the cartilage; (3) in addition, increased local concentration of cytokines, such as insulin-like growth factor (IGF-1), transforming growth factor (TGF- *β*), and interleukin, would also regulate chondrocytes’ catabolism and contribute to cartilage degeneration (Funck-Brentano & Cohen-Solal, 2015; Lajeunesse, 2002).

### Pathological lesions of cartilage and bone in CI model and potential mechanisms

The mechanism of the naturally-occurring OA induced by collagenase injection is different from that of the secondary OA. Collagen is a major component of connective tissue and widely exists in the matrix of ligaments, articular cartilage, and joint capsule. The type I collagen exists mainly in ligaments, while type II collagen exists mostly in cartilage (Nimni, 1991). The reason of cartilage damage in CI group was probably: (1) collagenase II directly destroys the collagen and proteoglycan directly in the cartilage matrix when injected into the knee joint cavity (Yeh et al., 2008); (2) the metabolism of chondrocytes was affected as a result of cartilage matrix damage, and these chondrocytes accelerate cartilage degeneration in turn (Van Osch et al., 1996); (3) the damage process in cartilage matrix produces sufficient degradation products (MMP-13), and these products promote further degradation, resulting in matrix mineralization and death of chondrocytes (Poole et al., 2003).

The absorption of subchondral bone is probably secondary to cartilage lesion, we made this speculation based on our results: (1) collagenase II is unable to break down the type I collagen contained in the bone; (2) at the 6th week, IHC results showed a more significant decrease of type II collagen in the CI group than that in the ACLT group, but less increase of Runx2 in CI group than that in ACLT; (3) quantitative analysis and reconstruction of micro-CT showed that subchondral bone reconstruction occurred later in CI model than that in ACLT model. Therefore, we supposed that cartilage damage occurred first in CI model. The mechanism of subchondral bone changes was probably as follows: (1) the rough surface and defects of cartilage directly increase the local stress on the subchondral bone (loading area); (2) after cartilage destruction, a series of reparative and destructive cytokines are produced locally, which is important for initiation of bone remodeling. These cytokines can get across the tidemark and trigger bone loss (Bondeson et al., 2006; De Croos et al., 2006).

### Clinical treatment and prevention for primary and secondary OA

Presently, treatment goals for OA are mainly to minimize both pain and functional loss. It involves pharmacologic therapies and arthroplasty.

Nonsteroidal anti-inflammatory drugs (NSAIDs) are recommended drugs for symptom relief in OA. Glucosamine and chondroitin sulphate, which are naturally constituents of articular cartilage proteoglycans, have also been widely used. Intra-articular injection of hyaluronic acid has been widely considered effective (Bhandari et al., 2017). However, these traditional and widely-used pharmacologic therapies are merely target on cartilage degradation. Because the initiating factor and pathogenesis may be different between naturally-occurring OA and secondary OA, whether the drugs target on articular cartilage or bone to should be considered.

Targets on bone remodeling are promising. As shown in micro-CT analysis, a transient osteoporosis, which means the resorption before the remodeling of subchondral bone, would occur at the early phase in both natural occurring and secondary OA models. Some previous reports have revealed that fracture of subchondral caused by osteoporosis would promote progression of OA (Horikawa et al., 2014; Osterberg et al., 2017); osteoporosis inhibition drugs, such as alendronate and calcitonin, would not only effectively inhibit bone resorption and subchondral bone remodeling, but also displayed chondral protective effects (Yeh et al., 2008; Botter et al., 2009; Bellido et al., 2011; Sondergaard et al., 2007). Some studies suggested that alendronate would even decrease the on incidence of OA in ovariectomy rats (Zhu et al., 2013).

Therefore, early use of osteoporosis inhibition drugs would be effective for the treatment of OA. For trauma-related secondary OA model, because the cartilage degeneration occurs secondary to bone resorption, early use of osteoporosis inhibition drugs seems to be more important. For naturally-occurring OA model, because the articular cartilage degeneration is the initiating factor of OA, cartilage protection drugs seems to be more important. In addition, early use of both cartilage repair and osteoporosis inhibition drugs would probably be more effective than use any of them alone, in either naturally-occurring or trauma-related secondary OA.

### Limitations

First, it is noticeable that the dosage and concentration of collagenase probably exert great influence on the simulation results. Secondly, it would probably be more close to naturally-occurring OA by using smaller dosage and more injection times.

We mainly confirmed the dose based on other studies (Seo et al., 2016; Nirmal et al., 2017; Adães et al., 2015; Adães et al., 2014), and we also did a pilot study. Till now, there are a few studies adopting collagenase to induce osteoarthritis in rats and made subsequent studies, and the dosage and frequency varied. For example, one study used a dose of 85U type II collagenase for first injection, and a booster injection after 4 days. Four weeks after first injection, the authors found obvious osteoarthritic histopathological features (Seo et al., 2016). Another study used two injections of 50 U into the knee joint to induce osteoarthritis (Nirmal et al., 2017). Some studies used two injections of 250 U and 500 U (Adães et al., 2015; Adães et al., 2014), and even one-time injection of 1.5k U (Jeong et al., 2018). We have made a pilot study using an injection of 20 U and 200 U, and found no significant osteoarthritic changes in the former group after 2 weeks. Therefore, in this study, we adopt a dose of 200 U to induce osteoarthritis.

Because naturally-occurring osteoarthritis is a chronic degenerative disease, a smaller and more frequent dosage should probably be closer to the state. For example, a daily injection of 10U for a few weeks may be better to induce osteoarthritis than a large dose of injection, which causes more rapid and acute damages. Unfortunately, no studies have noted this point.

## Conclusions

In this study, we successfully induced two rat models to simulate two clinical types of OA, and compared their different histopathology changes in articular cartilage and subchondral bone at the early stage. Results showed different pathogenic mechanisms in the two models at the early phase. Cartilage damage is probably secondary to subchondral bone remodeling in the ACLT model, while subchondral bone remodeling is probably secondary to cartilage degeneration in the CI model. The two different animal models can not only reveal mechanisms of the two types of OA, but also provide more insights on potential treatment and future research on OA.

##  Supplemental Information

10.7717/peerj.8934/supp-1 Data S1 Raw data of histological assessment, micro-CT and immunohistochemical staining between ACLT and CI models (A) Modified mankin scores. (B) BV/TV. (C) Tb.Sp. (D) Tb.Th. (E) TRAP positive staining of osteoclasts. (F) Immunohistochemical staining of Col II. (G) Immunohistochemical staining of MMP13. (H) Immunohistochemical staining of Runx2

## Additional Information and Declarations

### Data Availability

The following information was supplied regarding data availability: The raw data is available in File S1.
